# Supplementary material for: The effect of intrapartum prolonged oxygen exposure on fetal metabolic status: secondary analysis from a randomized controlled trial
Source: Front Endocrinol (Lausanne). 2023 Jun 27;14:1204956. doi: 10.3389/fendo.2023.1204956 (PMC10335765; doi:10.3389/fendo.2023.1204956)
Supplement: Supplementary file 3 [file DataSheet_3.zip › Result-X101SC21092977-Z01-J001-B1-42/4.MetDiffAnalysis/O.vs.A/O.vs.A_pos_corr.pdf]

O.vs.A

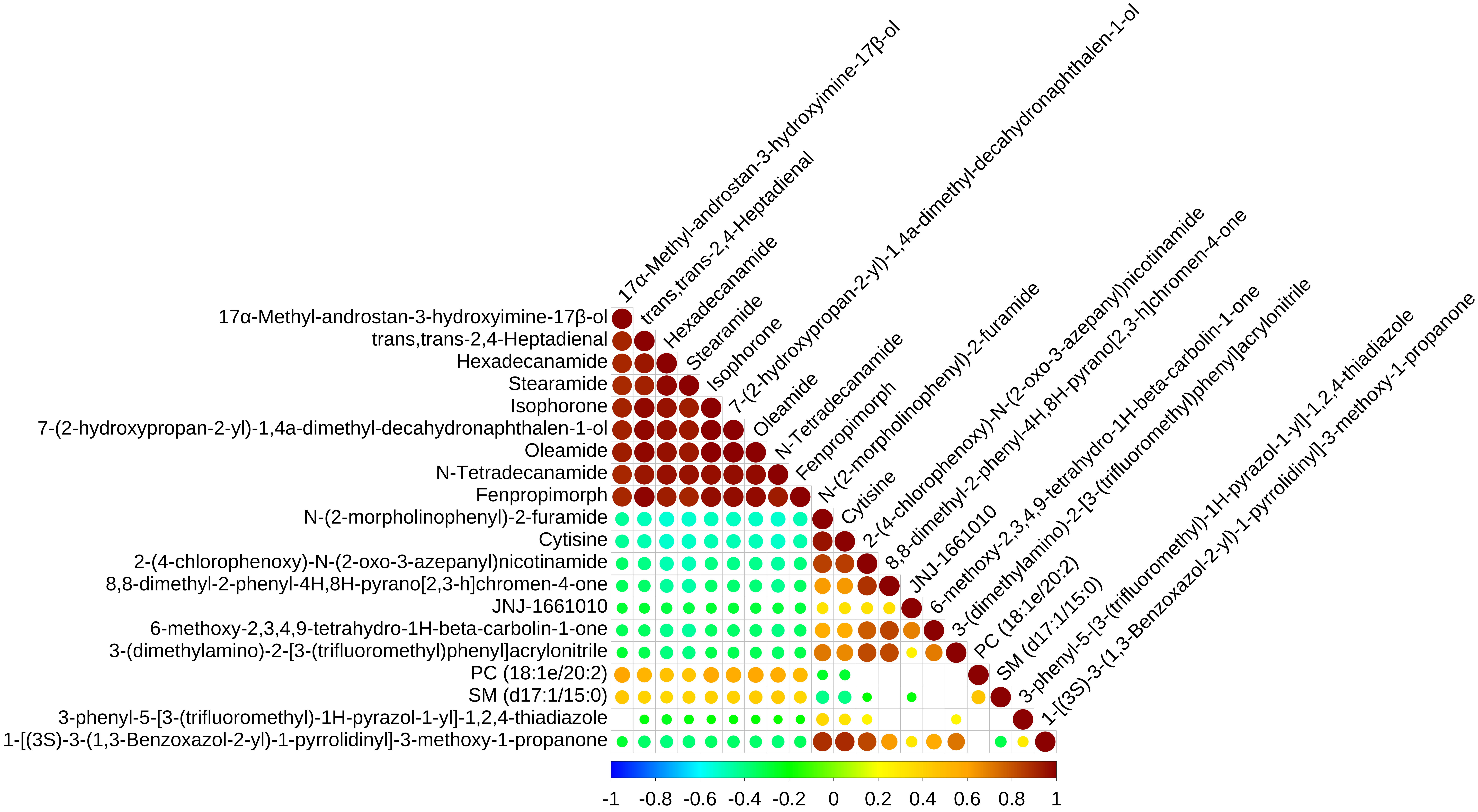

17α-Methyl-androstan-3-hydroxyimine-17β-ol
trans,trans-2,4-Heptadienal
Hexadecanamide
Stearamide
Isophorone
7-(2-hydroxypropan-2-yl)-1,4a-dimethyl-decahydronaphthalen-1-ol
Oleamide
N-Tetradecanamide
Fenpropimorph
N-(2-morpholinophenyl)-2-furamide
Cytisine
2-(4-chlorophenoxy)-N-(2-oxo-3-azepanyl)nicotinamide
8,8-dimethyl-2-phenyl-4H,8H-pyrano[2,3-h]chromen-4-one
JNJ-1661010
6-methoxy-2,3,4,9-tetrahydro-1H-beta-carbolin-1-one
3-(dimethylamino)-2-[3-(trifluoromethyl)phenyl]acrylonitrile
PC (18:1e/20:2)
SM (d17:1/15:0)
3-phenyl-5-[3-(trifluoromethyl)-1H-pyrazol-1-yl]-1,2,4-thiadiazole
1-[(3S)-3-(1,3-Benzoxazol-2-yl)-1-pyrrolidinyl]-3-methoxy-1-propanone
-1
-0.8
-0.6
-0.4
-0.2
0
0.2
0.4
0.6
0.8
1
